# Aggressive Recurrent Bilateral Chylothorax Secondary to Follicular Lymphoma in an HIV-Positive Patient: A Case Report and Clinical Implications

**DOI:** 10.7759/cureus.107289

**Published:** 2026-04-18

**Authors:** Amritpal S Jagra, Ruhma Ali, Alan Klukowicz, Hari O Sharma, Richard Miller

**Affiliations:** 1 Internal Medicine, Saint Michael's Medical Center, Newark, USA; 2 Pulmonary and Critical Care Medicine, Saint Michael's Medical Center, Newark, USA; 3 Intensive Care Unit/Critical Care, Hackensack Meridian Ocean Medical Center, Manahawkin, USA; 4 Intensive Care Unit, Saint Michael's Medical Center, Newark, USA

**Keywords:** follicular lymphoma, hiv, immunocompromised, opportunistic infections, recurrent bilateral chylothorax

## Abstract

Chylothorax is an infrequent form of pleural effusion, primarily associated with trauma or cancer. Bilateral chylothorax resulting from follicular lymphoma is especially rare, even more so in immunocompromised patients like those with HIV. Such cases can present diagnostic challenges by resembling infectious conditions.

A 52-year-old male with stable HIV presented with worsening shortness of breath and was found to have bilateral pleural effusions. While initial assessments prioritized infectious causes, pleural fluid analysis demonstrated significantly elevated triglycerides, establishing the diagnosis of chylothorax. Imaging revealed widespread lymph node enlargement, and a biopsy confirmed advanced follicular lymphoma. After diagnosis and placement of a portacath, the patient was lost to follow-up and later returned with recurrent bilateral chylothorax.

This case demonstrates a rare and aggressive manifestation of follicular lymphoma, ordinarily a slow-growing cancer, in an immunocompromised patient. Pleural effusions in individuals with HIV are often presumed infectious, which can delay the detection of underlying malignancies. Healthcare providers should include lymphoproliferative disorders in the workup of persistent pleural effusions, regardless of infectious risk factors, to ensure prompt oncologic referral. This case contributes to the scarce literature on chylothorax from follicular lymphoma and underscores the importance of a broad diagnostic perspective in immunocompromised patients.

## Introduction

Chylothorax, defined by the presence of chyle (a milky, triglyceride-rich lymphatic fluid) within the pleural cavity, is an uncommon form of pleural effusion, an accumulation of fluid between the layers of tissue lining the lungs and chest cavity. Most frequently, chylothorax arises due to trauma, surgical intervention, or, less commonly, malignancy [1]. Among cancers, lymphoma, particularly non-Hodgkin variants, is a recognized but rare cause, and follicular lymphoma is seldom responsible [2]. Bilateral chylothorax, meaning involvement of both pleural cavities, is even more unusual and typically occurs in advanced disease with widespread mediastinal (central chest) or retroperitoneal (abdominal) lymphadenopathy [2]. Recurrent chylothorax in the context of follicular lymphoma is extremely rare, with only a few cases described in the literature [3].

This case details a 59-year-old Portuguese woman with a history of HIV, hepatitis C, bipolar disorder, and substance use disorder who presented with new bilateral pleural effusions later identified as chylothorax. Imaging revealed a retroperitoneal mass with marked lymphadenopathy, and pathology confirmed follicular lymphoma. Remarkably, she experienced recurrent bilateral chylothorax within one month of diagnosis, a highly unusual and poorly understood occurrence in follicular lymphoma [3]. The rapid recurrence of effusions, despite the typically low-grade nature of the malignancy, suggests unusually aggressive disease behavior and highlights limitations in prognostic tools such as FLIPI in predicting such atypical courses [4].

This patient’s comorbidities, particularly HIV and hepatitis C, added complexity to both diagnosis and management. HIV is a recognized risk factor for lymphoma, likely due to chronic immune activation and decreased immune surveillance [5]. Coinfection with hepatitis C can increase the risk of liver toxicity from chemotherapy and further complicate care [6]. In addition, her bipolar disorder and substance use posed significant psychosocial challenges, as reflected by missed oncology appointments. These factors are increasingly acknowledged as critical influences on cancer outcomes, with evidence linking psychiatric illness and substance abuse to increased mortality and reduced treatment adherence [7,8].

This report aims to highlight the clinical significance and real-world implications of recurrent bilateral chylothorax in a patient with follicular lymphoma, emphasizing the challenges in diagnosis and management. Our objective is to raise awareness about the importance of considering malignant etiologies in patients presenting with bilateral pleural effusions, particularly in those with risk factors for lymphoma. By sharing this case, we underscore the potential for atypical and aggressive presentations of otherwise indolent malignancies, and we seek to encourage further research into the mechanisms, prognosis, and optimal management of recurrent chylothorax.

## Case presentation

The patient is a 59-year-old Portuguese woman with a medical background that includes hyperlipidemia, HIV managed with antiretroviral therapy, hepatitis C, polysubstance use (heroin and cocaine), depression, anxiety, bipolar disorder, and osteoarthritis. She came to the hospital reporting three weeks of exertional shortness of breath, retrosternal chest tightness, and a dry cough. She mentioned having had flu-like symptoms about a month earlier and received a 5-day course of azithromycin. She denied recent travel or contact with sick individuals. She smoked half a pack of cigarettes daily for 30 years and was adherent to her HIV therapy with Biktarvy (bictegravir/emtricitabine/tenofovir alafenamide) with a recent CD4+ count of 238 and an undetectable viral load.

**Figure 1:**
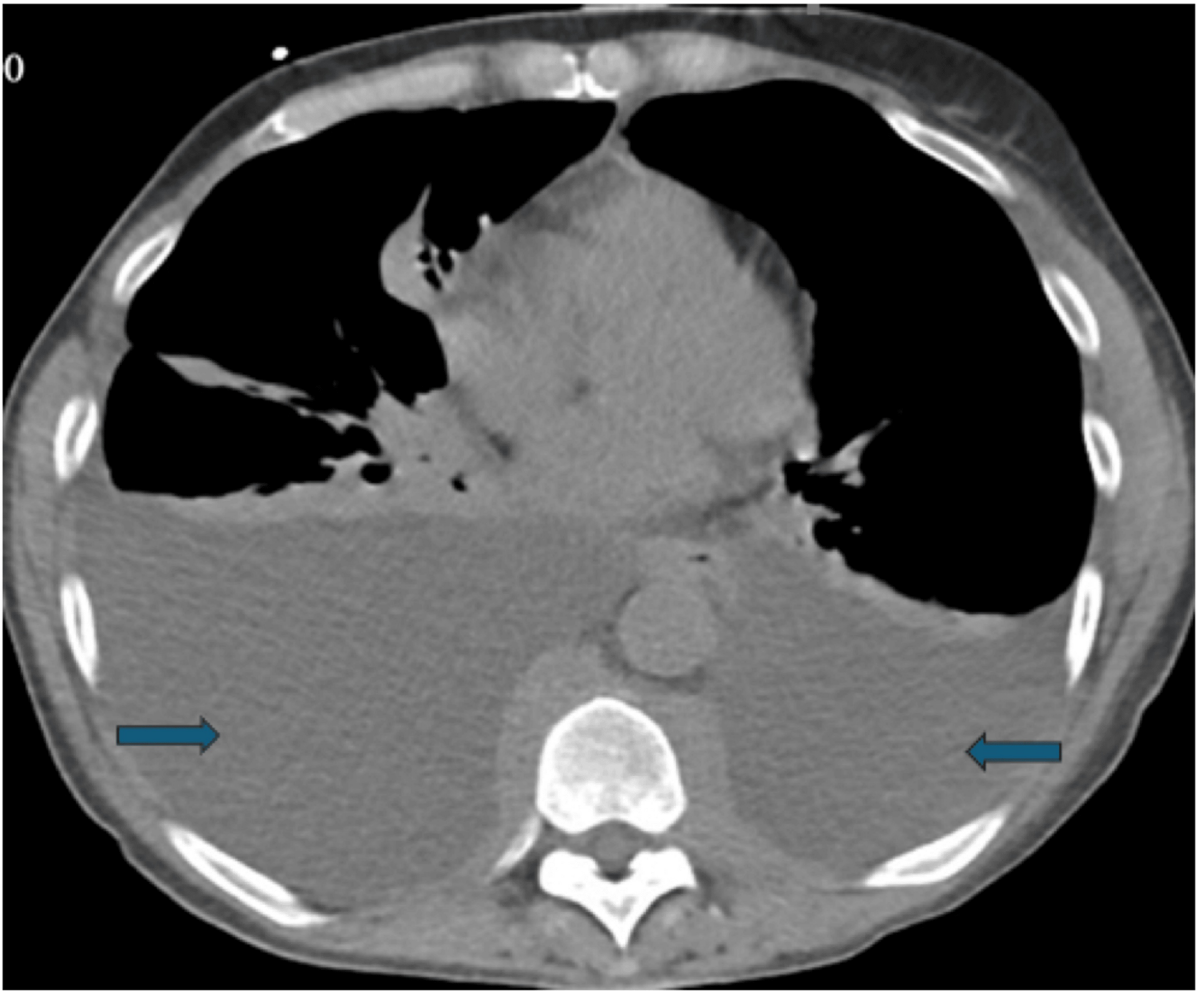
Axial CT of the thorax demonstrating bilateral pleural effusions (arrows), more pronounced on the right, with associated compressive atelectasis of the lower lobes.

**Figure 2:**
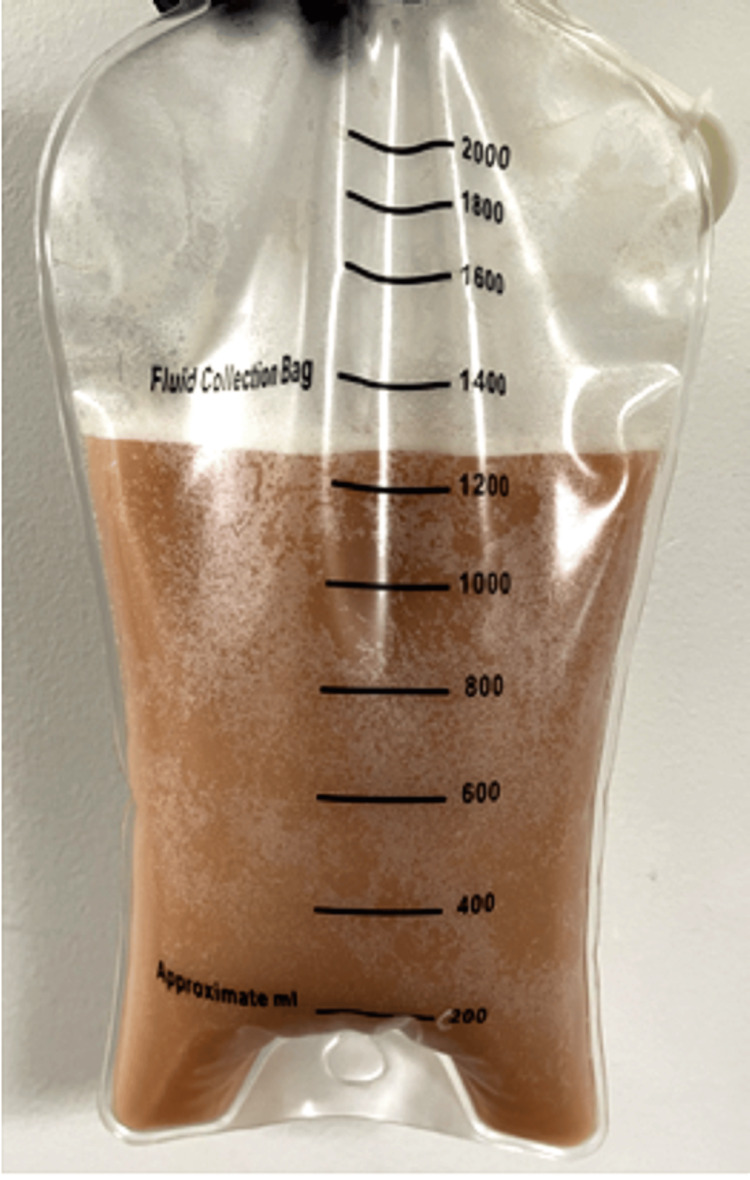
Milky pleural fluid in collection bag, suggestive of chylous effusion (chylothorax).

A CT angiogram of her chest was performed to simultaneously evaluate for pulmonary embolism and parenchymal pathology, offering higher diagnostic utility than HRCT or standard CECT in the acute setting. This showed massive bilateral pleural effusions along with significant retrocrural and upper retroperitoneal lymphadenopathy (Figure 1). Thoracentesis yielded milky fluid, which tested triglycerides at > 110 mg/dL and showed negative cytology (Figure 2).

**Figure 3:**
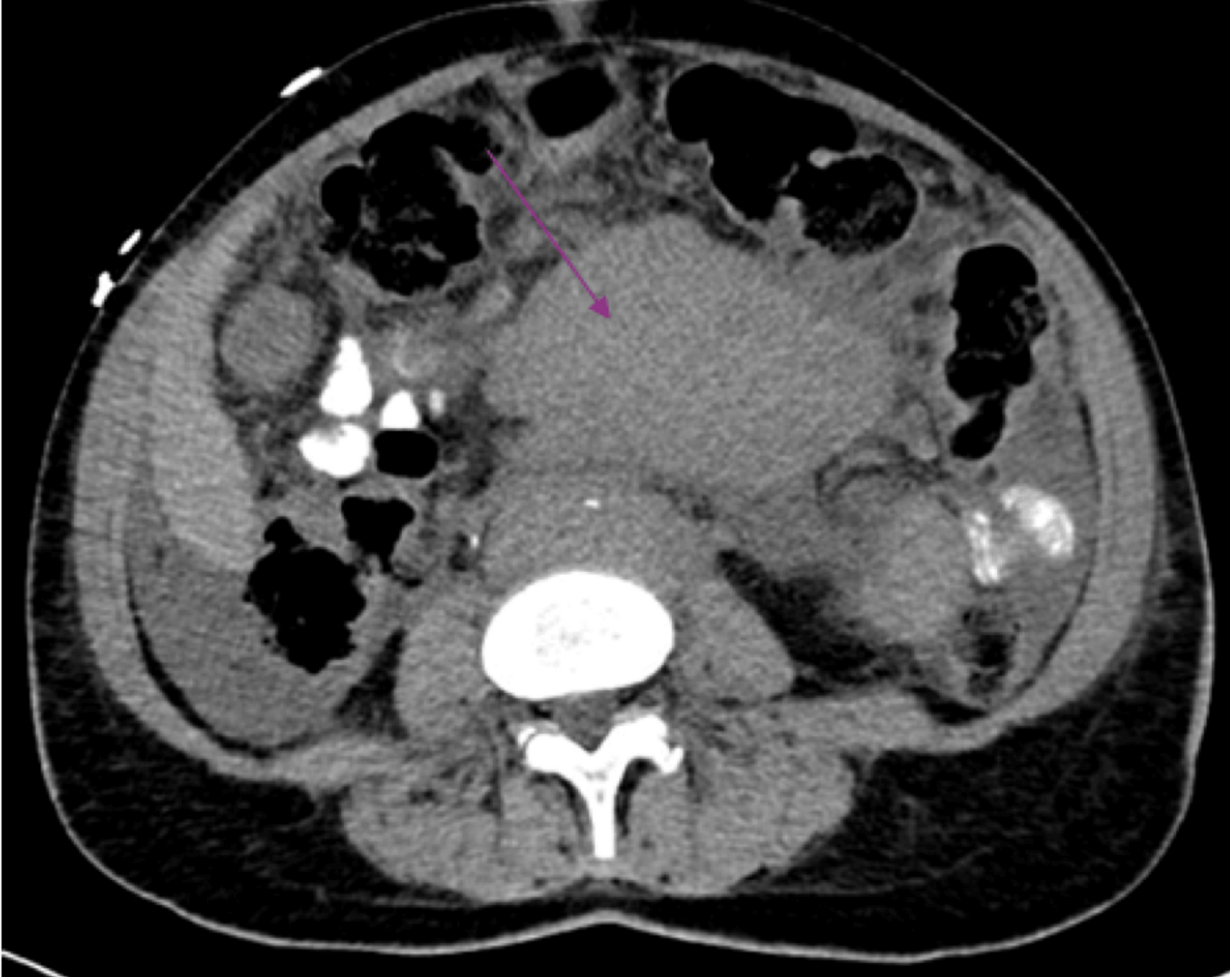
Contrast-enhanced axial CT image highlighting a bulky, heterogeneous abdominal mass (arrow) producing significant displacement of adjacent bowel structures.

Further imaging of the abdomen and pelvis found a large mid-abdominal soft tissue mass (measuring 7.6 × 11.6 × 15.9 cm) accompanied by bulky retroperitoneal lymph node enlargement (Figure 3). An IR-guided core needle biopsy of this mass revealed stage IV, grade II follicular lymphoma involving both intra-abdominal lymph nodes and the pleura. FISH (fluorescence in situ hybridization) analysis identified BCL6 and BCL2 rearrangements, and her FLIPI-1 score was 3, indicating high risk.

**Figure 4:**
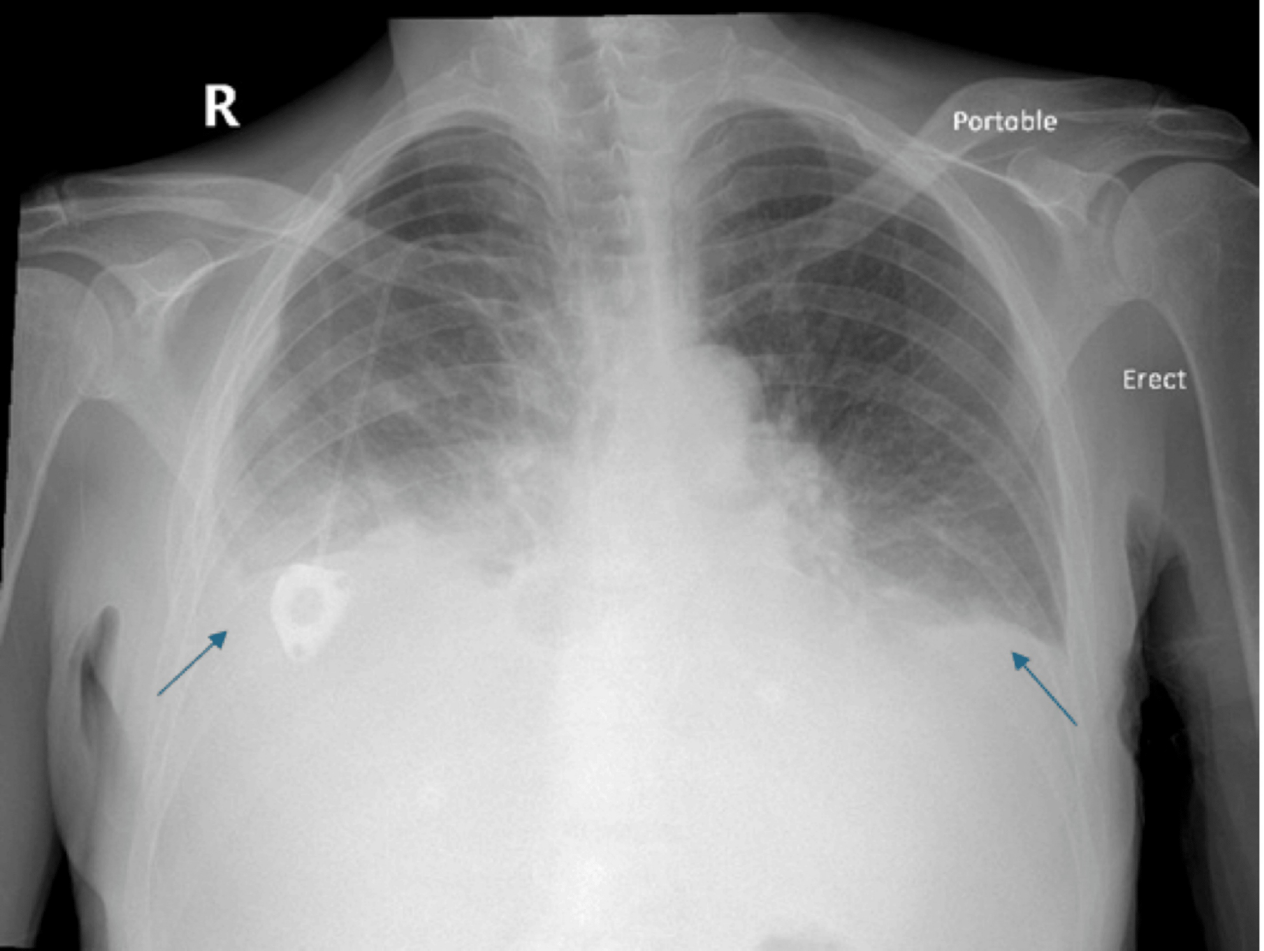
Follow-up chest X-ray one month after the initial presentation demonstrated recurrent bilateral pleural effusions (arrows).

A month afterward, she returned with shortness of breath upon exertion. Due to missed oncology appointments, she had not yet begun chemotherapy. Chest X-ray showed persistent, large bilateral pleural effusions, necessitating another thoracentesis (Figure 4).

## Discussion

This report of recurrent bilateral chylothorax in a middle-aged woman with HIV and chronic hepatitis C, ultimately found to have follicular lymphoma, showcases a rare and important manifestation of an otherwise slow-growing lymphoproliferative disorder in an immunocompromised patient. Although lymphoma can cause chylothorax, bilateral cases, especially from follicular lymphoma, are extremely rare. HIV complicates the case further by adding layers of chronic immune activation, suppression, and increased cancer risk.

Chylothorax generally arises from damage or blockage of the thoracic duct or central lymphatics, most commonly following trauma, surgery, or cancer. While aggressive lymphomas like diffuse large B-cell and Hodgkin lymphoma are well-known malignant causes, follicular lymphoma, a typically indolent B-cell non-Hodgkin lymphoma, rarely results in chylous effusions, especially on both sides. The bilateral chylothorax in this patient likely stemmed from widespread retroperitoneal and mediastinal lymphadenopathy, causing excessive chyle leakage and recurrent pleural fluid buildup. Such patterns are seldom described in the literature, highlighting the need for clinicians to recognize and thoroughly investigate these unusual presentations.

In this patient, the diagnosis was delayed because the initial evaluation focused on infectious causes, a common and understandable pitfall when assessing pleural effusions in individuals with HIV. While opportunistic infections are indeed a major concern in this population, this approach can inadvertently delay consideration of malignant etiologies. Recent studies indicate that HIV-related lymphomas frequently mimic infections both radiographically and clinically, contributing to diagnostic delays and poorer outcomes [9]. Notably, our findings demonstrate that even indolent lymphomas such as follicular lymphoma can present abruptly and atypically in the setting of immune dysregulation, underscoring the need for a more nuanced and context-driven diagnostic approach.

This case prompts a critical re-examination of how chronic immune alterations from HIV may modify the behavior of follicular lymphoma. Although follicular lymphoma is classically indolent, emerging evidence suggests that immunosuppression can precipitate a more aggressive and unpredictable disease course. In the present case, the rapid development of bilateral chylothorax and recurrent effusions may reflect a shift in the underlying biology of the lymphoma, potentially influenced by chronic inflammation, impaired immune surveillance, and persistent viral coinfections such as hepatitis C. These observations raise important questions about how immune status and comorbid infections interact to shape the clinical manifestations and progression of lymphoproliferative disorders.

These findings also highlight a critical gap in the oncologic and pulmonary literature. The manifestations of pleural and thoracic complications arising from indolent lymphomas in immunocompromised hosts remain poorly characterized. While the malignant effusions associated with aggressive, HIV-related lymphomas are well-documented, similar presentations in follicular lymphoma are seldom reported [10]. This paucity of data has significant clinical implications, as under-recognition of such atypical presentations can directly contribute to delayed diagnoses, suboptimal oncologic management, and inferior patient outcomes. There is a pressing need for more comprehensive research and reporting on these rare but consequential disease patterns.

Regarding treatment, the patient was scheduled to receive standard immunochemotherapy for follicular lymphoma, in accordance with current guidelines recommending full-dose regimens for HIV-positive individuals [11]. However, due to significant psychosocial challenges, including her comorbid bipolar disorder and substance use, she was ultimately lost to follow-up shortly after diagnosis. As a result, the outcome in terms of lymphoma response could not be assessed. This underscores the crucial impact of psychosocial factors on treatment adherence and highlights the importance of comprehensive support systems for patients facing complex medical and psychiatric burdens.

Overall, this case highlights the importance of a multidisciplinary strategy to ensure early detection of underlying cancer, prompt initiation of systemic treatment, and tailored support to address the wider challenges faced by patients with both cancer and infectious diseases. As more people with chronic viral infections live longer, clinicians should be alert to non-AIDS-defining cancers and their potential for unusual and aggressive clinical presentations.

## Conclusions

This report describes a rare presentation of recurrent bilateral chylothorax associated with follicular lymphoma in a patient with HIV infection. The case highlights the diagnostic complexity of bilateral pleural effusions and underscores the importance of considering lymphoproliferative disorders when chylothorax is identified. While causality between immune suppression and disease behavior cannot be definitively established from a single case, the observed clinical course raises the possibility that underlying immunocompromise may influence the presentation or progression of otherwise indolent lymphomas.

This case supports the need for timely diagnostic evaluation and early oncologic consideration in patients with HIV presenting with atypical or recurrent pleural effusions. It also emphasizes the importance of multidisciplinary care, including attention to social and follow-up barriers, which may impact outcomes. Further studies are needed to better define how immune status affects the natural history and prognosis of indolent lymphomas and whether risk stratification models should be adapted for patients living with HIV.

## References

[1] Soto-Martinez M, Massie J (2009). Chylothorax: diagnosis and management in children. Paediatr Respir Rev.

[2] Doerr CH, Allen MS, Nichols FC 3rd, Ryu JH (2005). Etiology of chylothorax in 203 patients. Mayo Clin Proc.

[3] Kosar F, Ergun D, Gunduz H Recurrent chylothorax due to follicular lymphoma: A case report and review of the literature. Case Rep Hematol.

[4] Solal-Céligny P, Roy P, Colombat P (2004). Follicular lymphoma international prognostic index. Blood.

[5] Kotecki N, Penel N (2016). Inappropriate dose of multitargeted tyrosine kinase inhibitors: the original sin. Curr Opin Oncol.

[6] Engels EA, Pfeiffer RM, Landgren O, Moore RD (2011). Immunologic and virologic predictors of AIDS-related non-Hodgkin lymphoma in the HAART era. J Acquir Immune Defic Syndr.

[7] Krebber AM, Buffart LM, Kleijn G (2014). Prevalence of depression in cancer patients: a meta-analysis of diagnostic interviews and self-report instruments. Psychooncology.

[8] Pirl WF, Greer JA, Traeger L (2012). Depression and survival in metastatic non-small-cell lung cancer: effects of early palliative care. J Clin Oncol.

[9] Zhu M, Chang Y, Fan H (2023). Primary pulmonary intravascular large B‑cell lymphoma misdiagnosed as pneumonia: Four case reports and a literature review. Oncol Lett.

[10] Jiang Y, Xie W, Hu K (2013). An aggressive form of non-Hodgkin's lymphoma with pleural and abdominal chylous effusions: a case report and review of the literature. Oncol Lett.

[11] Fukumoto A, Terao T, Kuzume A (2022). Management of lymphoma-associated chylothorax by interventional radiology and chemotherapy: a report of five cases. Int J Hematol.

